# Supplementary material for: Prospective Analysis of Prevalence, Trajectories of Change, and Correlates of Cannabis Misuse in Older Adolescents from Coastal Touristic Regions in Croatia
Source: Int J Environ Res Public Health. 2019 Aug 15;16(16):2924. doi: 10.3390/ijerph16162924 (PMC6721106; doi:10.3390/ijerph16162924)
Supplement: Supplementary file 1 [file ijerph-16-02924-s001.pdf]

Supplementary table

Table S1. Descriptive statistics for variables obtained at baseline and follow-up

	Baseline				Follow up			
	Nonusers		Misusers		Nonusers		Misusers	
	F	%	F	%	F	%	F	%
Gender								
male	226	49.13	120	65.22	192	48.00	154	63.11
female	234	50.87	64	34.78	208	52.00	90	36.89
Environment								
Rural	92	20.00	132	71.74	84	21.00	184	75.41
Urban	368	80.00	52	28.26	316	79.00	60	24.59
Individual sport participation								
Yes	99	21.52	27	14.67	72	18.00	54	22.13
Quit	184	40.00	110	59.78	178	44.50	116	47.54
No	177	38.48	47	25.54	150	37.50	74	30.33
Team sport participation								
Yes	111	24.13	35	19.02	84	21.00	62	25.41
Quit	221	48.04	105	57.07	212	53.00	114	46.72
No	128	27.83	44	23.91	104	26.00	68	27.87
Experience sport								
Never participated	64	13.91	20	10.87	48	12.00	36	14.75
< 1 year	41	8.91	15	8.15	40	10.00	16	6.56
2-5 years	139	30.22	75	40.76	136	34.00	78	31.97
> 5 years	216	46.96	74	40.22	176	44.00	114	46.72
Sport competitive result								
Never involved/competed	158	34.35	66	35.87	146	36.50	78	31.97
Competed locally	200	43.48	84	45.65	180	45.00	104	42.62
Competed Nationally	86	18.70	34	18.48	60	15.00	60	24.59
Competed International	14	3.04	0.0	0.00	12	3.00	2	0.82

Socioeconomic status								
Under average	10	2.17	8	4.35	14	3.50	4	1.64
Average	387	84.13	161	87.50	342	85.50	206	84.43
Above average	61	13.26	15	8.15	42	10.50	34	13.93
Paternal education								
Elementary school	16	3.48	6	3.26	10	2.50	12	4.92
High school	216	46.96	90	48.91	202	50.50	104	42.62
College level	124	26.96	56	30.43	106	26.50	74	30.33
University degree	100	21.74	32	17.39	78	19.50	54	22.13
Maternal education								
Elementary school	10	2.17	0	0.00	10	2.50	0	0.00
High school	255	55.43	117	63.59	234	58.50	138	56.56
College level	95	20.65	57	30.98	80	20.00	72	29.51
University degree	96	20.87	10	5.43	72	18.00	34	13.93
